# A clinical study of pattern and factors affecting outcome in Nigerian patients with advanced heart failure

**Published:** 2007-07

**Authors:** OB Familoni, TO Olunuga, BW Olufemi

**Affiliations:** Department of Medicine, Olabisi Onabanjo University Teaching Hospital, Sagamu, Nigeria; Department of Medicine, Olabisi Onabanjo University Teaching Hospital, Sagamu, Nigeria; Department of Epidemiology and Medical Statistics, College of Medicine, University of Ibadan, Ibadan, Nigeria

## Abstract

**Background:**

Advanced heart failure (AHF) accounts for about 25% of all cases of heart failure in Nigeria and is associated with a high mortality rate.

**Objective:**

To undertake a clinical study of the pattern and outcome of AHF in our hospitalised patients and to determine the parameters associated with mortality and survival in these patients.

**Method:**

Eighty-two patients with AHF were studied between January 2003 and December 2005. Baseline blood chemistry and haemodynamics were determined. A congestion score, including orthopnoea, elevated jugular venous pressure, oedema, ascites and loud P2, was derived as well as a low perfusion score. Mortality was computed and risk estimated using the Pearson coefficient and log-ranking test. Cox regression analysis was used to identify the predictors of survival.

**Results:**

AHF accounted for 43.6% of all hospitalised heart failure patients, with a total mortality of 67.1%. Hypertension was the commonest cause of AHF. The parameters associated with increased mortality rates included age (*r* = 0.671; *p* = 0.02), presence of atrial fibrillation (*r* = 0.532; *p* = 0.045) and estimated glomerular filtration rate (*r* = 20.486, *p* = 0.04). The majority of patients (54.8%) were in the ‘wet and cold’ congestion category. The congestion score correlated with mortality. The indices of survival included lower age, lower systolic blood pressure, being literate and lower congestion score.

**Conclusion:**

AHF was common in our cohorts of hospitalised heart failure patients and it was associated with a high mortality rate.

## Summary

The clinical syndrome of heart failure has been described as a ‘final pathway’ – the endpoint of a myriad diseases that affect the heart.^1^ It affects nearly five million people in the United States^2^ and up to three million people in the United Kingdom.^3^ Although community-based figures are rare in Africa, it is known that cardiovascular diseases are increasingly being recognised as important causes of morbidity and mortality in developing countries.^4^ The peak incidence of heart failure in patients remains in the fifth decade in Africa.^5^

Advanced heart failure (AHF), which has been defined as symptoms limiting daily life [New York Heart Association (NYHA) class III or IV] despite previous therapy with angiotensin converting enzyme inhibitors (ACEI), diuretics, digoxin and more recently β-adrenergic receptor blockers when tolerated, 6 is said to account for about 25% of the diagnosed heart-failure population.^2^ Patients with left ventricular ejection fraction (LVEF) less than 25% are said to have AHF if they conform to previous therapy as stated above.^2^

The features of AHF are essentially dominated by those related to congestion: orthopnea, jugular venous distension, ankle oedema, recent weight gain or the increased need for diuretics. These are reflections of elevated filling pressure.^7^ It has been found that survival ranges from 80% at two years for patients rendered free of congestion to less than 50% at six months for patients with refractory symptoms.^2^ Furthermore, Nohria *et al.*, recognising the prime place of congestion in the classification and prognosis of heart failure, suggested a bedside assessment that emphasises features of congestion, such as oedema and raised jugular pressure versus features of low perfusion, such as cool forearm and narrow pulse pressure, in order to group patients into categories of ‘warm and dry’, ‘warm and wet’, ‘cold and dry’ and ‘cold and wet’. This has been shown to guide therapy and prognosis in AHF.^8^,^9^

Clinical assessment therefore remains a useful, perhaps sufficient predictor for most cases of AHF.^2^ Echocardiography, which measures among other things the LVEF, is an essential tool in assessing the status and severity of heart failure, but heart failure remains a clinical diagnosis. Echocardiography is not necessarily the gold standard, as functional status and prognosis bear little relation to the LVEF alone.^3^,^10^

Other factors that have been shown to affect outcome in AHF include NYHA class, LVEF and left ventricular dimensions, co-morbid status, age, atrial fibrillation and anaemia.^02^ Renal dysfunction as manifested by estimated glomerular filtration rate (eGFR) has been shown to be a very strong predictor of mortality, stronger than NYHA class and LVEF.^10^,^11^ Biochemical markers including serum sodium, plasma urea and creatinine have additional prognostic values.^12^ Neurohormone tests, particularly B-type natriuretic peptides, are said to out-perform all other tests including systolic ejection fractions and other comprehensive echocardiographic measures in the Tei index.^13^,^14^

In a developing economy like ours with limited facilities, survival from AHF could be poor. Whereas previous workers have studied pattern and outcome of heart failure in our environment, we are not aware of studies focusing specifically on AHF. We therefore studied the pattern and outcome of AHF in our environment and also considered some of the factors that affect mortality in this cohort of patients.

## Patients and methods

Olabisi Onabanjo University Teaching Hospital, Sagamu is situated in a semi-urban area in the south-west region of Nigeria, midway between the industrial cities of Lagos and Ibadan. It serves a population of about 500 000 people. Cardiac patients are admitted to the medical wards containing 65 beds.

This was a prospective study of all patients admitted to the medical wards between January 2003 and December 2005 who met the clinical definition of AHF^6^ or had LVEF less than 25% despite adequate therapy.^2^ All the patients gave informed consent to participate in the study. All patients with a diagnosis of heart failure were reviewed within 24 hours by authors OBF/TOO for clinical features and drug management. Those who were in heart failure were divided into two groups, fulfilling or not fulfilling the criteria of AHF.

Those who had AHF had baseline investigations of blood count, blood chemistry and chest X-ray. GFR was estimated using the Cockcroft–Gault equation which has been validated in Nigerian patients as representative of the real determined GFR.^15^ A proportion of the patients had echocardiography performed as available using the ALOKA SSD-1 machine and American Society of Echocardiography (ASE) methods of leading-edge to leading-edge convention. The LVEF was automatically determined by the machine from a two-dimensional generated M-mode tracing of the right and left ventricular diameters. Echocardiography was also useful in confirming clinical aetiology in this group of patients.

A congestion score was derived; modified after Nohria *et al.*^2^ One point each was allocated to orthopnea, elevated jugular venous pressure, oedema, ascites and loud P2. The higher the score, the greater the evidence of congestion. The congestion score was also compared with a perfusion score made up of pulse pressure less than 40 mmHg, cool forearm and/or head, serum sodium less than 120 mEq/l, eGFR less than 40 ml/min and increasing daytime sleepiness. A patient was adjudged to have significant congestion and/or low perfusion if he scored at least three of the maximum five points. Mortality was also compared with the congestion score.

Literacy was defined as ability to read and write, with a formal school education of at least six years. The patients were followed up until death, or in out-patients on discharge and until the end of the study.

## Statistical analysis

Data were analysed using the statistical package for social sciences (SPSS 12). Descriptive statistics were computed for continuous variables and frequency tables for categorical variables were generated. All data were reported as mean ± SD and frequencies were expressed as percentages. The Pearson coefficient was used to test the correlation between relevant variables. The Kaplan−Meier curves were used to obtain estimation of survival rates, while the log-rank test was used to compare survival rates. The Cox proportional-hazard regression model was used to identify predictors of survival outcome. Prognostic variables were evaluated using the Chi-square test. Level of significance was put at *p* < 0.05.

## Results

There were 188 patients admitted for heart failure during the study period, of which 82 (43.6%) satisfied the criteria for AHF. Table 1 shows the baseline characteristics of the 82 patients. The mean age of presentation was in the sixth decade and the majority was male. Total mortality over the 36-month study period was 67.1%. Thirty-nine (70.9%) of the male patients died compared with 16 (59.2%) of the female. Although there was a greater tendency of death in males, this did not reach statistical significance (*p* = 0.124; χ^2^ = 2.371). Mortality in illiterates was 22/32 patients (68.8%) compared with 33/50 (66.0%) in those who were literate (*p* = 0.919; χ^2^ = 0.169).

**Table 1:** Baseline Characteristics Of All 82 Patients

*Parameter*	*n (%)*
Age (years)	57.6 ± 15.9
Gender (male)	55 (67.1)
Literate	50 (61.0)
NYHA class III	57 (69.5)
IV	25 (30.5)
Clinical aetiology of AHF	
Hypertension	36 (43.4)
Dilated cardiomyopathy	23 (28.0)
Rheumatic heart disease	8 (9.8)
Ischaemic heart disease	7 (8.5)
Cor pulmonale	3 (3.7)
HIV cardiomyopathy	2 (2.4)
Endomyocardial fibrosis	2 (2.4)
Infective endocarditis	1 (1.2)
BMI (kg/m^2^)	25.7 ± 3.4
SBP (mmHg)	131.7 ± 30.4
DBP (mmHg)	82.3 ± 22.8
PCV (%)	34.9 ± 7.0
Serum urea (mg/dl)	52.1 ± 37.9
Serum creatinine (mg/dl)	1.5 ± 1.2
eGFR (ml/min)	68.5 ± 25.5
Atrial fibrillation	17 (20.7)
LVEF (%)	22.3 ± 5.1
Mortality	55 (67.1)

The commonest cause of AHF was hypertension, occurring in almost half of all the patients, followed by dilated cardiomyopathy. The aetiology of the heart failure was not related to mortality (*p* = 0.235; χ^2^ = 9.257). The older the patient the greater the risk of dying from AHF (*r* = 0.671; *p* = 0.02) and also the presence of AF (*r* = 0.532; *p* = 0.045) conferred increased risk of death. eGFR was associated with mortality (*r* = 20.486; *p* = 0.041). When patients with packed-cell volume (PCV) less than 30% were compared with those above 30%, log-rank statistics revealed 5.43; *p* = 0.02. Neither NYHA class nor LVEF correlated with mortality in this study.

Table 2 compares the evidence of congestion with low perfusion in the patients. All the patients had higher indices of congestion compared with low perfusion. There were 45 (54.8%) patients who were in the ‘cold and wet’ category, 34 (41.4%) in the ‘warm and wet’ and only three (3.7%) were in ‘cold and dry’ category. Congestion score correlated with mortality. There was almost twice the risk of death in those whose congestion score was greater than three, compared with those whose score was three and below (RR 1.53; *p* < 0.05).

**Table 2:** Clinical Characteristics Of All Patients

*Evidence of congestion*		*Evidence of low perfusion*	
Parameter	n (%)	Parameter	n (%)
Orthopnoea	80 (97.6)	Cool forearm/head	47 (57.3)
Oedema	75 (91.5)	Daytime sleepiness	32 (60.1)
Ascites	70 (85.4)	eGFR < 40 ml/min	27 (32.9)
Raised JVP	69 (84.1)	Pulse pressure < 40 mmHg	12 (14.6)
Loud P2	67 (81.7)	Serum Na < 120 mEq/l	11 (13.4)

Table 3 shows a comparison of the parameters in the 55 patients who died and the 27 patients who were alive at the end of the study. The variables that showed significance to mortality include congestion score (*p* = 0.007), perfusion score (*p* = 0.04), eGFR (*p* = 0.009) and age (*p* = 0.02). Patients who were in AF were also likely to die.

**Table 3:** Comparison Of The Clinical Parametres In Survivors And Patients Who Died

*Variable*	*Survivors (n 5 27)*	*Death (n 5 55)*	*p-value*
Age (years)	54.3 ± 18.3	65.6 ± 10.7	0.02
Male (n %)	16 (59.3)	39 (70.9)	0.12
Literate (n %)	17 (63.0)	33 (60.0)	0.87
NYHA class III	21 (77.8)	36 (65.0)	0.34
IV	6 (22.2)	19 (34.5)	0.16
BMI (kg/m^2^)	24.8 ± 6.3	27.2 ± 3.1	0.56
SBP (mmHg)	128.4 ± 18.6	142.6 ± 14.8	0.04
DBP (mmHg)	80.3 ± 18.5	85.5 ± 16.3	0.63
PCV (%)	33.8 ± 8.2	27.0 ± 1.4	0.30
Serum urea (mg %)	52.0 ± 10.8	53.4 ± 10.4	0.76
Serum creatinine (mg %)	0.9 ± 0.4	1.5 ± 0.7	0.06
Serum Na (mEq/l)	132.6 ± 6.6	124.8 ± 3.2	0.08
eGFR (ml/min)	75.5 ± 15.3	45.6 ± 18.6	0.009
AF (*n* %)	3 (11.1)	14 (25.5)	0.02
LVEF (%)	23.6 ± 6.2	18.8 ± 8.1	0.42
Congestion score > 3 (*n* %)	12 (44.4)	33 (60.0)	0.007
Perfusion score > 3 (*n* %)	11 (40.7)	27 (49.1)	0.04

Table 4 shows the result of the Cox proportional-hazard regression analysis. The β-coefficient of the regression values revealed that the younger age group (0.003) and being literate (20.248) were associated with a greater tendency to survival on a long-term basis. Patients with higher values of systolic blood pressure (0.002), congestion score (0.005), creatinine levels (0.272) and NYHA class (0.460) were associated with poorer survival; that is they had higher hazards of death and a worse prognosis. Only the congestion score reached statistical significance in this study.

**Table 4:** Result Of COX Proportional Hazard Regression Analysis

*Variable*	*β*	*Std error*	p*-value*	*Exp β*	*95% CI for exp β*
Age	0.003	0.016	0.856	0.997	0.996–1.029
Literate	–0.248	0.482	0.607	0.781	0.304–2.008
NYHA	0.236	0.510	0.664	1.266	0.446–3.440
SBP	0.002	0.008	0.809	1.002	0.986–1.018
Creatinine	0.272	0.793	0.732	1.312	0.227–6.209
Congestion score	0.005	0.019	0.012	1.007	0.976–1.021

Thirty-three (40.2%) patients had the aetiology of AHF confirmed by echocardiography; 16 with dilated cardiomyopathy, nine with hypertensive heart failure and four patients with rheumatic heart disease. The diagnosis in the two patients with EMF and one with infective endocarditis were made with echocardiography. One patient with acute myocardial infarction also had an echocardiogram recorded. The mean LVEF in patients with dilated cardiomyopathy was 18% and that in hypertensive patients 32%.

## Discussion

AHF accounted for nearly half of the patients with heart failure during the study period. This was higher than the average of 25% quoted in the literature.^2^ This can be explained by the fact that this was a hospital-based study where one would expect AHF to be more frequent, and also due to late presentation to hospital, which has been variously observed in cardiac patients in our environment.^16^ Heart failure, being a progressive disease, could have reached advanced stages by the time these patients presented. This might partly explain the high mortality in this series; more than the 50% annual mortality reported in the literature. Our patients presented a decade later than patients with heart failure in another African series.^5^ Older age group was associated with mortality and poor prognosis in this study as in other observational studies.

Hypertension was the commonest cause of AHF in this study and it continues to be the leading cause of all heart failure on our continent.^5^ Significantly, rheumatic heart disease is becoming less common as a cause of either heart failure in general or AHF. It is understandable that rheumatic heart disease will be less widespread as a cause of AHF in adults because these patients tend to present in their early twenties and also tend to die before the peak age of presentation of AHF in the sixth decade.

Compromised renal function, as demonstrated by reduced eGFR, is associated with mortality in these heart failure patients. This has been shown to be a strong predictor of mortality in AHF. In a previous work, we have shown that aggravated renal dysfunction is common in our patients with AHF undergoing intense diuretic therapy and that it is associated with increased mortality.^17^

Atrial fibrillation occurred in 20.7% of our patients, comparable with the 23.8% in the study by Hillege *et al.*,^10^ which correlated with the mortality rate in this study. Atrial fibrillation by loss of atrial contribution to diastolic filling and decreased diastolic filling time in rapid AF is likely to decrease cardiac output and make heart failure worse.

The congestion score showed a strong association with mortality in this study. There were more patients in the ‘cold and wet’ category in this study compared with the series quoted by Nohria *et al.*^8^ (55 vs 28%). This might be explained by late presentation in our patients and the larger proportion of patients with dilated cardiomyopathy, with its attendant poor perfusion characteristics, in addition to congestion in these patients. Previous workers have noted a poorer prognosis in patients with congestion at rest. It has also been shown that if the congestion score can be decreased during out-patient visits, the prognosis could be improved. Unfortunately, the general observation in our patients was a need for diuretic dose increase at out-patient clinics due to increased congestion. This is compounded by the fact that many defaulted on drugs and clinic appointment.

Neither NYHA class nor LVEF was associated with mortality in this study, unlike in the work by Hillege *et al.* where the parameters correlated, albeit weakly, with mortality.^10^ Although NYHA class did not correlate with mortality in this study, when the Cox proportional hazard regression analysis was considered, it was found that a higher NYHA class (III vs IV, as in this study) was associated with poorer survival.

In spite of its prolonged use and universal applicability, the shortcomings of the NYHA classification is well recognised, one of which is the fact that it does not make for identification and screening of patients at risk.^1^ This has led to the recent classification by the American College of Cardiology and American Heart Association, which graded patients from stage A – at risk without structural heart disease, to stage D – end-stage symptoms of heart failure that are refractory to standard treatment.^18^ Stage D corresponds to the NYHA class III/IV and AHF. Other workers have employed symptom classifications, which are useful for their local environments.^19^ We are not aware of any classification specifically developed for our locality.

Another parameter associated with higher hazard of death was systolic blood pressure (SBP). The paradigm shift to SBP has shown that it is more associated with adverse cardiovascular events than diastolic BP.^20^ Higher values of serum creatinine and reduced eGFR are signs of poor renal function and this has been associated with poor survival in patients with AHF. This was also the experience in this study.

The conclusion of this study is that AHF was common in the cohort of our patients with heart failure and the mortality rate was high. Hypertension remained the commonest cause of AHF. The mortality rate was increased by older age group, lower packed-cell volume and deranged renal function. The majority of our AHF patients belonged to the ‘cold and wet’ congestion group with its attendant high mortality rate. Long-time survival was associated with lower values of SBP, lower creatinine values and being literate.

The limitations of this study include the relatively small number of patients, which naturally detracts from the power to draw conclusions. Autopsy was not performed to actually determine the cause of death; the study assuming all-cause mortality. It is also noteworthy that not all the patients had echocardiograms. Although this was basically a clinical study, an echocardiogram is still desirable for aetiological diagnosis, haemodynamics and follow-up.

It is recommended that larger longitudinal and perhaps multicentre studies be undertaken to properly define the scope of the problem in our community. Greater attention should be paid to the control of hypertension, particularly SBP, which most studies show is more difficult to control than diastolic BP. Health education, including earlier presentation in hospital, is advocated.
